# Donor CD47 controls T cell alloresponses and is required for tolerance induction following hepatocyte allotransplantation

**DOI:** 10.1038/srep26839

**Published:** 2016-05-27

**Authors:** Mingyou Zhang, Hui Wang, Shulian Tan, Nalu Navarro-Alvarez, Yang Zheng, Yong-Guang Yang

**Affiliations:** 1 First Hospital and Institute of Immunology, Jilin University, Changchun, Jilin, China; 2 Columbia Center for Translational Immunology, Columbia University, New York, NY, United States

## Abstract

CD47-deficient hepatocyte transplantation induces rapid innate immune cell activation and subsequent associated graft loss in syngeneic recipients. However, the role of donor CD47 in regulation of T-cell alloresponses is poorly understood. We addressed this question by assessing OVA-specific immune responses in mice following hepatocyte transplantation from CD47-competent or -deficient OVA-transgenic donors. Compared to sham-operated controls, intrasplenic transplantation of CD47-deficient OVA^+^ hepatocytes significantly accelerated rejection of OVA^+^ skin grafted 7 days after hepatocyte transplantation. In contrast, mice receiving CD47-competent OVA^+^ hepatocytes showed prolonged and even indefinite survival of OVA^+^ skin allografts. T cells from mice receiving CD47-deficient, but not CD47-competent, OVA^+^ hepatocytes showed significantly enhanced responses to OVA^+^ stimulators compared to sham-operated controls. In contrast to the production of tolerogenic cytokines (IL-4 and IL-10) in the recipients of CD47-competent hepatocytes, mice receiving CD47-deficient hepatocytes showed elevated production of IFN-γ and IL-1α. Moreover, significant expansion of myeloid-derived suppressor cells was detected in the recipients of CD47-competent hepatocytes, which was required for tolerance induction in these mice. Thus, donor CD47 plays an important role in the control of T-cell alloresponses and tolerance induction following hepatocyte transplantation. Our data also suggest that intrasplenic hepatocyte transplantation may provide a means to induce allograft tolerance.

A balance between the stimulatory and inhibitory signals regulates the innate immune cell activation. Signal regulatory protein-alpha (SIRPα) is an inhibitory receptor expressed on macrophages, and upon interaction with its ligand CD47, a member of the Ig superfamily, provides a ‘don’t eat me’ signal to macrophages, which is required for preventing phagocytosis of self hematopoietic cells^1^. Consistent with this premise, previous studies demonstrated that the inability of donor CD47 to functionally interact with the recipient SIRPα induces rapid rejection of xenogeneic hematopoietic cells by macrophages^2^,^3^. In a syngeneic model of mouse hepatocyte transplantation, we recently showed that the lack of CD47 expression on donor hepatocytes elicits innate immune cell activation and graft rejection^4^. However, the role of CD47 in allogeneic hepatocyte transplantation has not been studied.

Liver allografts have been shown to induce tolerance in both small and large animal models, as well as in patients^5^,^6^,^7^. Previous studies in rats showed that liver parenchymal cells play an important role in spontaneous liver-induced tolerance^8^ and consistently, hepatocyte transplantation leads to suppression of anti-donor immune responses^9^. Using a mouse model of hepatocyte allotransplantation, here we show that donor CD47 plays a critical role in controlling the development of anti-donor T cell responses and its expression is required for tolerance induction following transplantation of allogeneic hepatocytes. Hepatocyte transplantation from CD47-competent donors led to inhibition of anti-donor T cell responses and induction of allotolerance through a mechanism dependent on myeloid-derived suppressor cells (MDSCs). However, in contrast to the tolerogenic potential of CD47-competent hepatocytes, transplantation of CD47-deficient hepatocytes paradoxically enhanced anti-donor T cell responses.

## Results

### CD47 KO but not WT hepatocyte transplantation stimulates donor antigen-specific T cell responses

**Figure 1:**
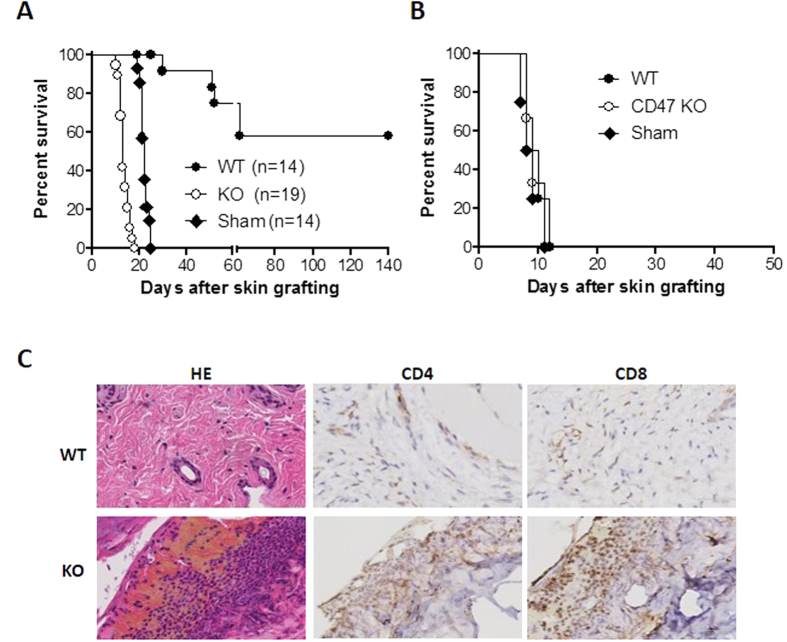
Skin allograft survival in mice receiving WT or CD47KO hepatocyte transplantation. WT B6 mice received sham-operation (Sham) or hepatocyte transplantation from WT or CD47KO OVA-Tg B6 donors, followed 7 days later by WT-OVA-Tg B6 mouse skin transplantation. Some recipient mice also received third-party (BALB/c) mouse skin grafts at the same time. (**A**) OVA-Tg mouse skin graft survival. (**B**) Third-party mouse skin graft survival. (**C**) OVA-Tg mouse skin grafts were harvested for histological analysis from the recipients of WT (top) or CD47KO (bottom) hepatocytes 13 days after skin transplantation. Shown are representative samples stained with HE and IHC (with anti-CD4 or anti-CD8).

To determine the role of donor CD47 in the development of anti-donor T cell responses, we grafted OVA-transgenic (Tg) B6 mouse skin onto wild-type (WT) B6 mice that received intrasplenic transplantation of hepatocytes from WT- or CD47KO-OVA-Tg B6 donors, or sham operation (controls) 7 days prior to skin grafting. All sham-operated controls rejected OVA-Tg mouse skin grafts between 19 and 25 days, with a median survival time (MST) of 21 days (Fig. 1A). Compared with the control recipients, transplantation of CD47KO-OVA-Tg mouse hepatocytes significantly (p < 0.0001) accelerated donor skin graft rejection. In these mice, OVA-Tg mouse skin grafts were rejected between 10 and 18 days, with a MST of 13 days. In contrast, mice receiving WT-OVA-Tg mouse hepatocytes paradoxically prolonged the survival of OVA-Tg B6 mouse skin grafts, with approximately 60% of the mice achieved long-term survival (>140 days). Unlike donor skin grafts, third-party mouse skin allografts were similarly rejected in all three groups (Fig. 1B). We also conducted repeat OVA-Tg B6 mouse skin grafting onto two mice with long-term skin graft survival 140 days after 1^st^ skin grafting. In one of these mice, the second graft survived up to 66 days and both the first and second grafts were rejected around similar times (at day 60 and day 66 post-second skin transplantation, respectively). In the second mouse, neither the first nor the second skin grafts were rejected during the observation period of 100 days after the second skin transplantation. However, acute rejection (on days 8 and 10, respectively) of third-party mouse skin allografts was seen in both animals. The data suggest the presence of an active mechanism to maintain donor-specific tolerance in mice receiving WT hepatocyte transplantation.

We also harvested skin grafts from the recipient mice 13 days after transplantation for histological examination. The hematoxylin and eosin (HE) staining revealed normal structure without inflammatory infiltration in the skin grafts from mice receiving WT-OVA hepatocytes, but severe tissue damage with massive inflammatory cell infiltration in the skin grafts from the recipients of CD47KO-OVA hepatocytes (Fig. 1C). Furthermore, immunohistochemistry (IHC) demonstrated an extensive CD4 and CD8 T cell infiltration in the skin grafts from CD47KO hepatocyte-transplanted mice, but not in those from WT hepatocyte-transplanted mice (Fig. 1C).

**Figure 2:**
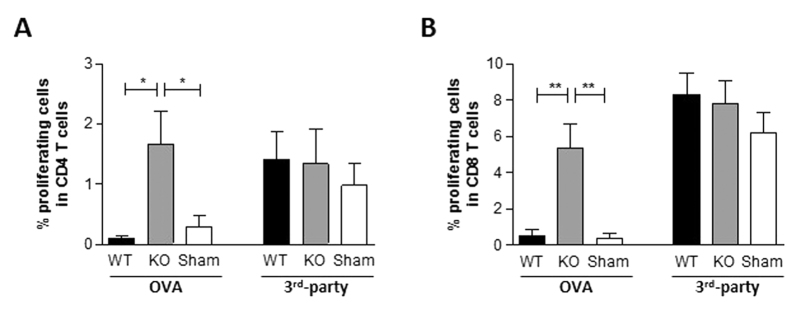
Stimulation of anti-donor T cell responses by CD47KO but not WT hepatocyte transplantation. Spleen cells were prepared from WT B6 mice 7 days after transplantation of WT or CD47KO OVA-Tg B6 hepatocytes, and analyzed for proliferative responses of T cells to OVA or 3^rd^-party alloantigen by CFSE dilution assay. Shown are percentages (mean ± SDs) of proliferating cells in gated CD4^+^ (**A**) and CD8^+^ (**B**) T cell populations. *p < 0.05, **p < 0.01.

Next, we measured T cell proliferative responses to OVA stimulation *in vitro*. Spleen cells were prepared from the recipient mice 7 days after hepatocyte transplantation, stained with CFSE, and co-cultured with irradiated (30 Gy) splenocytes from WT-OVA-Tg B6 mice or BALB/c mice (3^rd^ party) for 4 days. Flow cytometric (FCM) analysis revealed that T cells, including both CD4 (Fig. 2A) and CD8 (Fig. 2B) T cells, from mice receiving CD47KO-OVA-Tg mouse hepatocytes showed a significantly enhanced proliferation in comparison with those from WT hepatocyte-transplanted mice and sham-operated controls, whereas T cells from all 3 groups showed comparable proliferation in response to 3rd-party allogeneic stimulators. Together, these results indicate that CD47 expression on donor hepatocytes plays an important role in controlling the development of anti-donor alloresponses and is required for tolerance induction after hepatocyte transplantation.

### Distinct profiles of inflammatory cytokine production in mice following hepatocyte transplantation from WT vs. CD47KO mice

**Figure 3:**
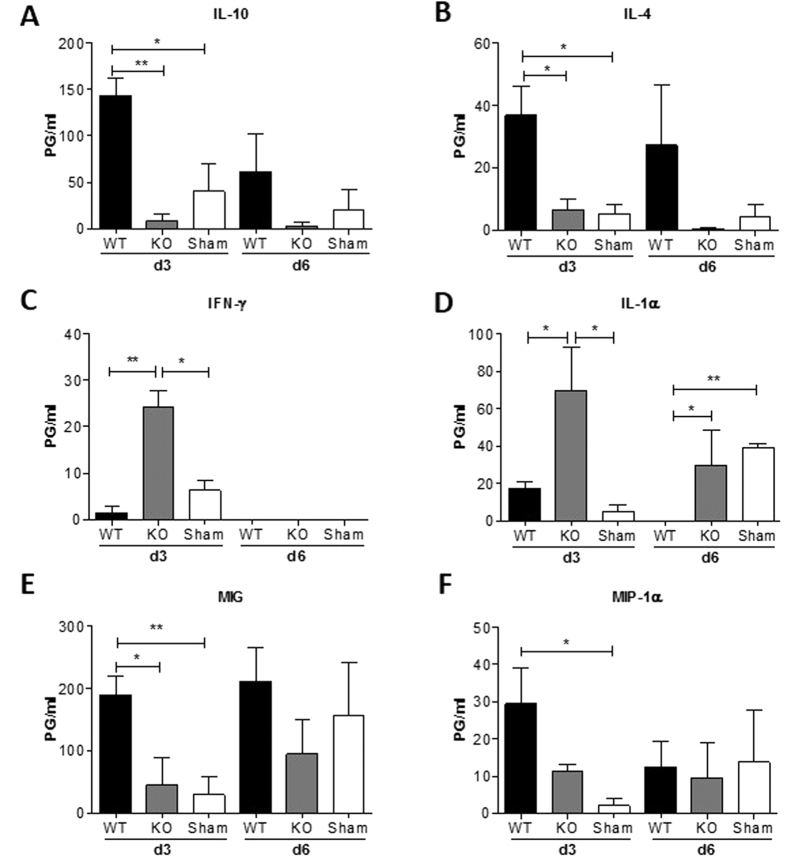
Plasma levels of cytokines and chemokines. Plasma was prepared from B6 mice receiving WT or CD47KO OVA-Tg B6 mouse hepatocytes 3 and 6 days after transplantation and analyzed for cytokines and chemokines by Luminex (n = 3 for each time point per group). Shown are plasma levels (mean±SDs) of IL-10 (**A**), IL-4 (**B**), IFN-γ (C), IL-1α (**D**), MIG (**E**) and MIPα (**F**). *p < 0.05, **p < 0.01.

We have shown that transplantation of CD47KO hepatocytes induces extensive innate immune cell activation (e.g., expansion of Mac-1^+^ cells) in syngeneic mice^4^. Expectedly, innate immune cell activation, as shown by a significant increase in Mac-1^+^ cells, was also detected in mice receiving CD47KO-OVA-Tg hepatocytes compared to those receiving WT-OVA-Tg hepatocytes and the sham-operated controls (Fig. S1). To further characterize the early inflammatory responses, plasma was collected 3 and 6 days after hepatocyte transplantation, and measured for the levels of an array of inflammatory cytokines and chemokines (see Methods). Mice receiving WT and CD47KO hepatocytes exhibited distinct profiles of cytokine and chemokine production, particularly at the early time point (day 3). WT hepatocyte transplantation led to the production of tolerogenic cytokines (IL-10 and IL-4) while CD47KO hepatocyte transplantation showed elevated production of pro-inflammatory cytokines (IFN-γ and IL-1α) (Fig. 3A–D). Furthermore, a significant increase in MIG and MIP-1α was seen the recipients of WT hepatocytes compared to those receiving CD47KO hepatocytes or sham-operated controls (Fig. 3E,F).

### MDSCs play an important role in tolerance induction by hepatocyte transplantation

**Figure 4:**
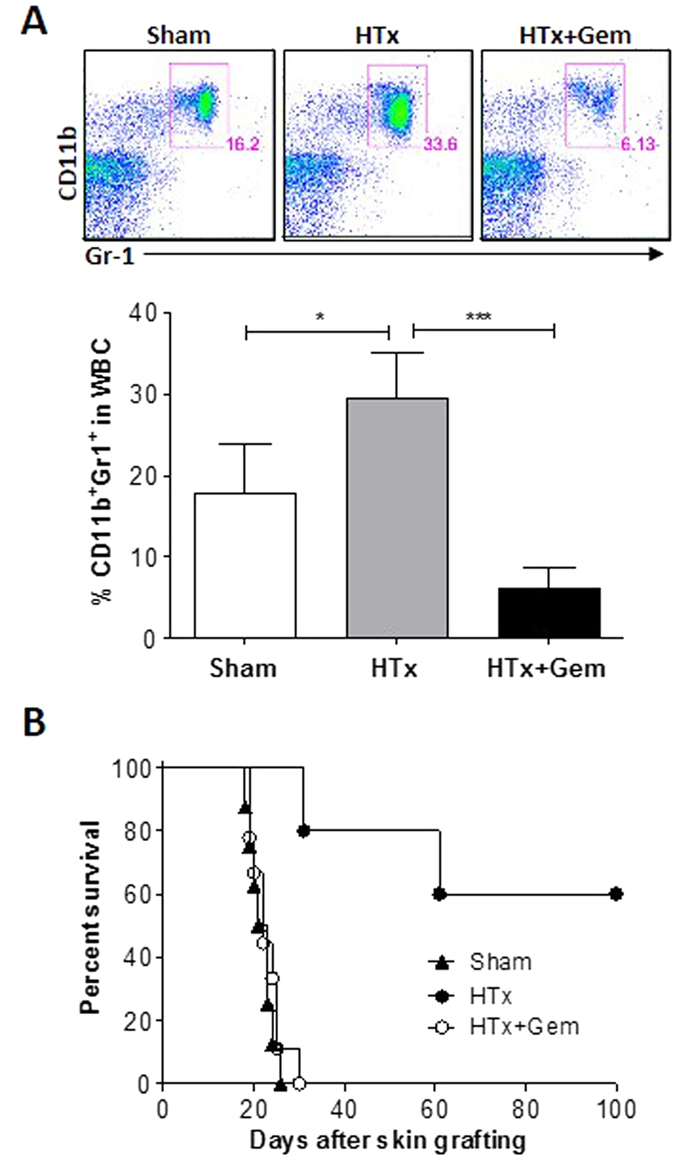
MDSC expansion contributes to tolerance induction by hepatocyte transplantation. B6 mice received sham operation (Sham) or transplantation of WT-OVA-Tg B6 mouse hepatocytes without (HTx) or with gemcitabine treatment (HTx + Gem), followed 7 days later by OVA-Tg mouse skin transplantation (n = 5–9/group). (**A**) Representative staining profiles (top) and percentages (mean ± SDs; bottom) of CD11b^+^ Gr1^+^ MDSCs in PBMCs analyzed by flow cytometry 2 days after hepatocyte transplantation. *p < 0.05, ***p < 0.001. (**B**) Skin graft survival.

MDSCs are a heterogeneous population of cells that expand during inflammation and infection. In mice, MDSCs are CD11b^+^ Gr1^+^^10^ and have been shown to contribute to tolerance induction^11^. In support of the role of MDSCs in tolerance induced by WT hepatocyte transplantation, a significant increase in the frequency of CD11b^+^ Gr1^+^ MDSCs was detected in mice receiving WT hepatocyte transplantation compared to the sham-operated controls (Fig. 4A). To further determine whether MDSCs are responsible for tolerance induced by WT hepatocyte transplantation, we compared the survival of skin allografts in recipient mice with or without MDSC depletion. Treatment with gemcitabine 1 day prior to hepatocyte transplantation led to a marked decrease in MDSCs (Fig. 4A), which is consistent with previous studies showing that gemcitabine can selectively deplete MDSCs in mice^10^,^12^. Importantly, MDSC depletion by gemcitabine completely abrogated tolerance in the WT hepatocyte-transplanted mice, in which OVA-Tg skin grafts were rejected in a similar time course as the sham-operated controls (Fig. 4B).

## Discussion

Liver is considered a tolerogenic organ^13^ and liver allografts may induce tolerance^6^. Both parenchymal and non-parenchymal cell components of the liver may contribute to its tolerogenic properties^8^,^13^. Here we showed that intrasplenic transplantation of WT hepatocytes induces robust tolerance, leading to durable donor skin allograft survival without additional immunosuppression. However, in contrast to the tolerogenic potential of WT hepatocyte transplantation, transplantation of CD47-deficient hepatocytes paradoxically stimulates anti-donor T cell responses, leading to accelerated donor skin graft rejection. Furthermore, the expansion of MDSCs was found to be required for tolerance induction by WT hepatocyte transplantation.

The efficacy of Treg cells in the induction of transplantation tolerance and prevention of allograft rejection has been well documented^14^,^15^. However, we found no correlation between the number of Treg cells and allograft survival, suggesting that tolerance induction by hepatocyte transplantation is predominantly mediated by other mechanisms, rather than induction of Treg cells (Fig. S2). MDSCs contribute to immunosuppression in both tumors and transplantation^11^. Previous studies have shown the association of MDSC accumulation within the blood and allografts with tolerance induction, and the potential of graft-infiltrating MDSCs to suppress effector T cell expansion^16^. Here we found that WT hepatocyte transplantation cannot improve donor skin survival in mice that were selectively depleted of MDSCs, demonstrating that MDSCs play an important role in tolerance induction by hepatocyte transplantation. It has been shown that the suppressive activity of MDSCs requires not only the factors that promote MDSC expansion but also the factors that activate MDSCs^17^. Reactive oxygen species (ROS) is also considered an important factor that contributes to the suppressive activity of MDSCs^17^. Considering the role of IL-4 in MDSC activation^17^ and the role of IL-10 in inducing ROS production of MDSCs^18^, the Th2 type response induced by hepatocyte allotransplantation is likely to contribute to the observed suppression of anti-donor T cell responses by MDSCs.

Our findings reveal an important role for CD47 expression on donor cells in controlling T cell responses following hepatocyte allotransplantation. CD47 is a ligand of SIRPα, an inhibitory receptor expressed on macrophages and thus, blocking CD47-SIRPα interaction results in phagocytosis^1^,^3^,^19^. Indeed, not only macrophages, SIRPα is also expressed by dendritic cells (DCs), and blocking CD47-SIRPα interaction promotes DC endocytosis^3^,^19^,^20^. Our previous studies have shown that intravenous injection of CD47-deficient allogeneic splenocytes induces rapid activation of recipient SIRPα^+^ CD8^-^CD11^hi^ DCs, which was associated with an augmented anti-donor T cell responses^20^. Consistent with this, CD47 blockade was recently reported to enhance the ability of DCs to stimulate T cell antitumor responses^21^. Thus, CD47 plays important roles in the modulation of both the innate and adaptive immune systems. Here we found that the lack of donor CD47 not only fails to induce tolerance, but also augments anti-donor T cell responses. Compared to the sham-operated controls, mice that received CD47KO hepatocytes showed accelerated donor skin rejection and enhanced pro-inflammatory cytokine production, whereas prolonged allograft survival and production of tolerogenic cytokines were observed in the recipients of WT hepatocytes. Interestingly, the recipients of WT hepatocytes had a significantly increased early production of CXCL9 (MIG) and CCL3 (MIP-1α) compared to those receiving CD47KO hepatocytes. These data are somewhat contradictory to previous studies showing that increased CXCL9 production is associated with enhanced alloresponses^22^,^23^,^24^. Although CCL3 might be upregulated during allograft rejection^24^, it was also reported to induce T cell apoptosis^25^. Clearly, further studies are needed to understand the role of these chemokines in hepatocyte transplantation-induced tolerance.

As an alternative to orthotopic liver transplantation, hepatocyte transplantation represents an attractive strategy for treatment of acute liver failure and inherited metabolic disorders^26^,^27^,^28^,^29^. Unfortunately, the scarcity of allogeneic liver donors limits the utilization of this therapy. The use of hepatocytes from other species, i.e., xenotransplantation, represents untapped potential to solve this pressing clinical problem. This study, along with our previous research^2^,^3^,^4^,^20^, provides unequivocal evidence that the interspecies incompatibility in CD47 may induce both innate and adaptive immunity-mediated xenograft rejection.

## Methods

### Animals

Six- to eight-week-old female wild-type (WT), CD47KO and OVA-Tg mice on the C57BL/6 (B6) background were purchased from the Jackson laboratory. CD47KO-OVA-Tg B6 mice were generated by cross-breeding CD47KO B6 mice with OVA-Tg B6 mice. These mice were used as the recipients or donors for hepatocyte and skin transplantation as detailed in the text. Protocols involving animals were approved by the Columbia University Institutional Animal Care and Use Committee, and all animal experiments were performed in accordance with the protocols.

### Hepatocyte isolation and transplantation

Primary mouse hepatocytes were isolated by a two-step collagenase digestion method, as previously reported^30^. Hepatocyte viability evaluated by trypan blue staining was routinely >90%. Hepatocyte transplantation was conducted by direct injection of 1.5 × 10^6^ hepatocytes into the lower pole of the recipient spleen. The splenic artery was clamped for about 2 minutes to avoid immediate passage of hepatocytes into the portal vein, the infusion site was ligated, and the abdomen was sutured. In some experiments, the recipient mice were depleted of MDSCs by intravenous injection of gemcitabine (100 mg/Kg; LC Laboratories, Woburn, MA) 1 day prior to hepatocyte transplantation.

### Skin transplantation

Full thickness skin flaps were prepared from the tail of the donor mice and placed on the dorsal wall of the recipient mice. The graft was secured by corner suture and carefully bandaged for 7 days. Skin graft survival was followed by daily visual inspection for the first 3 weeks and once every 2 days thereafter. Grafts were defined as rejected when <10% of the graft remained viable.

### Histology and Immunohistochemistry (IHC)

Paraffin embedded skin sections were stained with hematoxylin and eosin (HE), or immunohistochemically using anti-mouse CD4 (bcam; ab51312) or CD8 mAb (DAKO; M7103), followed by staining with biotinylated secondary antibodies. The IHC procedure was performed using a highly sensitive Strept ABComplex/HRP detection system (Dako).

### T cell proliferation assays

The spleen from recipient mice was harvested 7 days after hepatocyte transplantation, and single cell suspension was prepared and used as responders. Spleen cells prepared from naive mice were used as stimulators. The responder cells were stained with fluorochrome 5,6-carboxyfluorescein diacetate succinimidyl ester (CFSE; Invitrogen), incubated without (medium controls) or with 30-Gy-irradiated stimulators for 4 days. Proliferation (defined as CFSE dilution) of the responder T cells was measured by flow cytometry, and presented as the net percentage (after subtraction of the medium control) of CFSE^low^ cells in gated CD4 or CD8 T cell population.

### Flow cytometric analysis of MDSCs

Peripheral blood was collected at the indicated time points, PBMCs were prepared and stained with fluorescence-labelled rat anti-mouse CD11b and anti-mouse Gr1 mAbs, or with rat IgG1 isotype control mAb (eBioscience). All samples were collected on a FACS (Fortessa, Becton Dickinson) and analyzed by Flowjo software (Tree Star).

### Analysis of cytokine and chemokine concentration

Plasma was collected from recipient mice at the indicated time points after hepatocyte transplantation, and the levels of cytokines and chemokines were measured with the Luminex^®^ Cytokine Mouse 20-Plex kit covering *basic* fibroblast growth factor, IL*-1*β, IL-10, IL-13, IL-6, IL-12, IL-17, macrophage inflammatory protein 1α (MIP-1α), granulocyte macrophage-colony stimulating factor (GM-CSF), monocyte chemoattractant protein-1 (MCP-1), IL-5, vascular endothelial growth factor (VEGF), IL-1α, INF-γ, tumor necrosis factor-α (TNF-α), IL-2, IFN-γ-induced protein 10 (IP 10), monokine induced by gamma interferon (MIG), keratinocyte-derived chemokine (KC), and IL-4. Data were analyzed with FlowCytomix Pro 2.4.

### Statistical Analyses

Graft survival data are presented as Kaplan-Meier survival curves and differences between groups were analyzed by the log-rank test using GraphPad Prism (version 6; San Diego, CA). Student’s t-test was used to calculate statistical difference in mean values between groups, and results are presented as mean ± SDs. A p value of < 0.05 was considered significant.

## Additional Information

**How to cite this article**: Zhang, M. *et al*. Donor CD47 controls T cell alloresponses and is required for tolerance induction following hepatocyte allotransplantation. *Sci. Rep.* **6**, 26839; doi: 10.1038/srep26839 (2016).

## Supplementary Material

Supplementary Information
