# Supplementary material for: Donor CD47 controls T cell alloresponses and is required for tolerance induction following hepatocyte allotransplantation
Source: Sci Rep. 2016 May 27;6:26839. doi: 10.1038/srep26839 (PMC4882503; doi:10.1038/srep26839)
Supplement: Supplementary Information [file srep26839-s1.doc]

# Donor CD47 controls T cell alloresponses and is required for tolerance induction following hepatocyte allotransplantation

Mingyou Zhang^1,2^, Hui Wang^2^, Shulian Tan^1,2^, Nalu Navarro-Alvarez^2^, Yang Zheng^1,*^, Yong-Guang Yang^1,2,*^

**Supplementary Information**


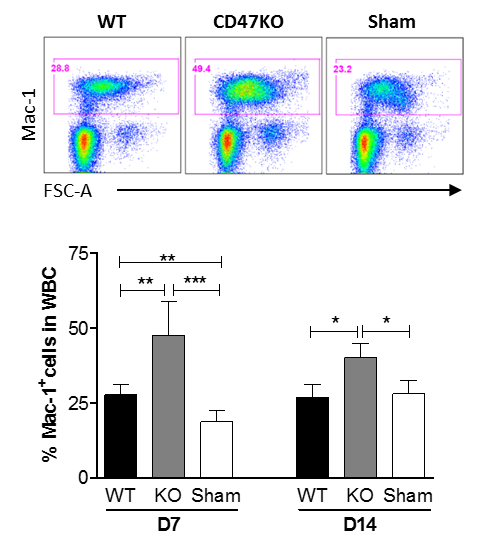


**Figure S1. Mac-1^+^ cell expansion in the recipients of WT vs. CD47KO hepatocyte transplantation.** B6 mice received sham-operation (Sham) or hepatocyte transplantation from WT or CD47KO OVA-Tg B6 donors, and WBCs were prepared 7 and 14 days later for flow cytometric analysis of Mac-1^+^ cells. Shown are representative staining profiles at day 7 (top) and percentages (mean±SDs) of Mac-1^+^ cells in WBCs at days 7 and 14 (bottom). * p<0.05, ** P<0.01, *** P<0.001.


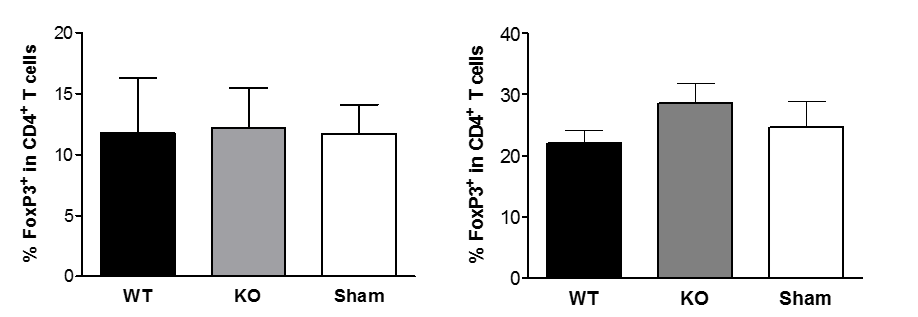


**Figure S2. Neither WT nor CD47KO hepatocyte transplantation induces changes in Treg cell numbers**. B6 mice received sham-operation (Sham) or hepatocyte transplantation from WT or CD47KO OVA-Tg B6 donors (n=4/group) and 7 days later, spleen and liver cells were prepared for analysis of CD4^+^Foxp3^+^ Treg cells. Shown are percentages (mean±SDs) of FoxP3^+^ cells in gated CD4^+^ T cell population in the spleen (left) and liver (right).
